# En-Mass Retraction of Maxillary Anterior Teeth with Severe Proclination and Root Resorption—A Case Report

**DOI:** 10.3390/diagnostics12051055

**Published:** 2022-04-22

**Authors:** Chenshuang Li, Wenlu Jiang, Shih-Chin Chen, Krisena Borenstein, Nipul Tanna, Chun-Hsi Chung, Won Moon

**Affiliations:** 1 Department of Orthodontics, School of Dental Medicine, University of Pennsylvania, Philadelphia, PA 19104, USA; nipul77@upenn.edu (N.T.); chunc@upenn.edu (C.-H.C.); 2 Department of Orthodontics, School of Dentistry, University of California, Los Angeles, CA 90095, USA; wlulujiang@gmail.com (W.J.); arielmay7227@gmail.com (S.-C.C.); krisena@gmail.com (K.B.); 3 Sunny Dental Clinic, Shanghai 310000, China; 4 Sunny Dental Institute for Clinical Research and Application, Beijing 100022, China; 5 The Forsyth Institute, Cambridge, MA 02142, USA; 6 Department of Orthodontics, Institute of Oral Health Science, Ajou University School of Medicine, Suwon-si 16499, Korea

**Keywords:** molar distalization, TADs, orthodontic root resorption, adult treatment

## Abstract

Molar distalization has been a validated method to correct dental sagittal relationships and create space to relieve mild to moderate crowding. In the current case report, an adult female patient had a mild skeletal Class III relationship and dental Class III molar relationship. Four premolars and one lower incisor were extracted during the previous two rounds of orthodontic treatments, and the maxillary anterior teeth were left with severe proclination and root resorption. Limited by the available teeth, extraction was not an option for her. Thus, molar distalization with TADs was the best option used in the treatment to address her chief complaint. In addition, a proper bite opening was performed to eliminate occlusion trauma. Utilizing the mid-palatal TADs, the maxillary central incisors were retracted 7.9 mm and retroclined 33 degrees, and the molar distalization was achieved as much as 8 mm. The cross-section slices of CBCT images confirmed the proper retraction of maxillary incisors and well-positioned roots in the alveolar bone. Moreover, the root resorption was not worsened from the treatment. Clinically, the maxillary anterior teeth were preserved esthetically and functionally. This case report illustrates that with proper diagnosis and treatment mechanics, significant tooth movement can be achieved even on extremely proclined maxillary incisors with severe root resorption.

## 1. Introduction

Correction of proclined and protrusive anterior teeth often requires space in the posterior region of the arch. For the mild anterior teeth retraction, posterior spaces can be created by molar distalization by a variety of inter- and intra-arch mechanics [1,2], while for moderate to severe anterior teeth retraction, premolar extraction is usually the choice to provide enough spaces [3]. In a scenario where extraction of four first premolars still does not provide enough space to retract the anterior teeth, additional molar distalization would be needed [4]. In some situations, extraction of another 4 premolars is performed (both premolars in each quadrant) [5], but it might lead to compromised and disturbed occlusal function. Additionally, anatomical limitations must be considered.

For molar distalization, both buccal or palatal placed temporary skeletal anchorage devices (TADs) have been utilized. The buccal TADs placement is more limited by surrounding anatomic structures than the palatal TADs placement. Therefore, for maxillary total arch distalization, mid-palatal TADs supported molar distalizers are often utilized [6].

On the other hand, orthodontic management of teeth with severe external root resorption is difficult and rarely reported [7], as the compromised crown/root ratio changed the stress distribution when regular orthodontic force was applied to the tooth and the root resorption may get worsened during the treatment [8].

This case report presented a young adult with severely proclined anterior teeth, missing four premolars and one mandibular incisor, and significantly compromised root length and alveolar bone support on maxillary incisors. The patient was treated successfully with total arch distalization with mid-palatal TADs.

## 2. Diagnosis and Etiology

A 24-years-old Asian female presented to the orthodontic clinic with a chief complaint “My previous orthodontist told me that I need orthognathic surgery, and the surgeon I consulted with referred me here for pre-surgical orthodontic treatment”. The patient was generally healthy and was not being treated for any medical illness, had no known drug allergies, and was not taking any medications. She denied any problems or pain associated with her temporomandibular joints (TMJ). The patient had a history of two rounds of orthodontic treatment, with three teeth extracted for the first round of orthodontic treatment and two teeth extracted for the second round. She stated that the previous orthodontist told her the “sticking-out” anterior teeth cannot be corrected orthodontically, and would need to be corrected by orthognathic surgery.

**Figure 1:**
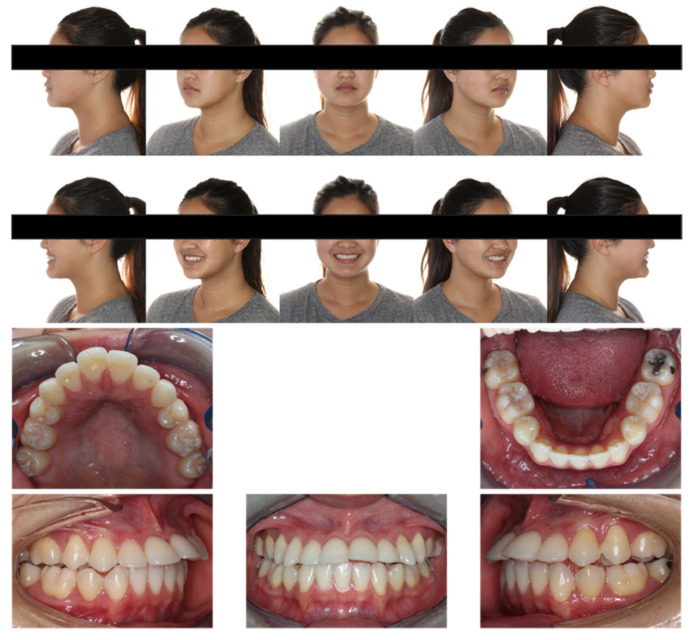
Pretreatment facial and intraoral photographs.

Extraoral examination (Figure 1) showed the following: anterior-posteriorly, the patient had a straight profile with a slightly protrusive upper lip; transversely, she had good facial symmetry, and normal buccal corridors on her smiling; vertically, she presented a mesofacial pattern. No mentalis strain or lip incompetence at rest was noted. She also exhibited a 90% incisor display on her smiling.

**Figure 2:**
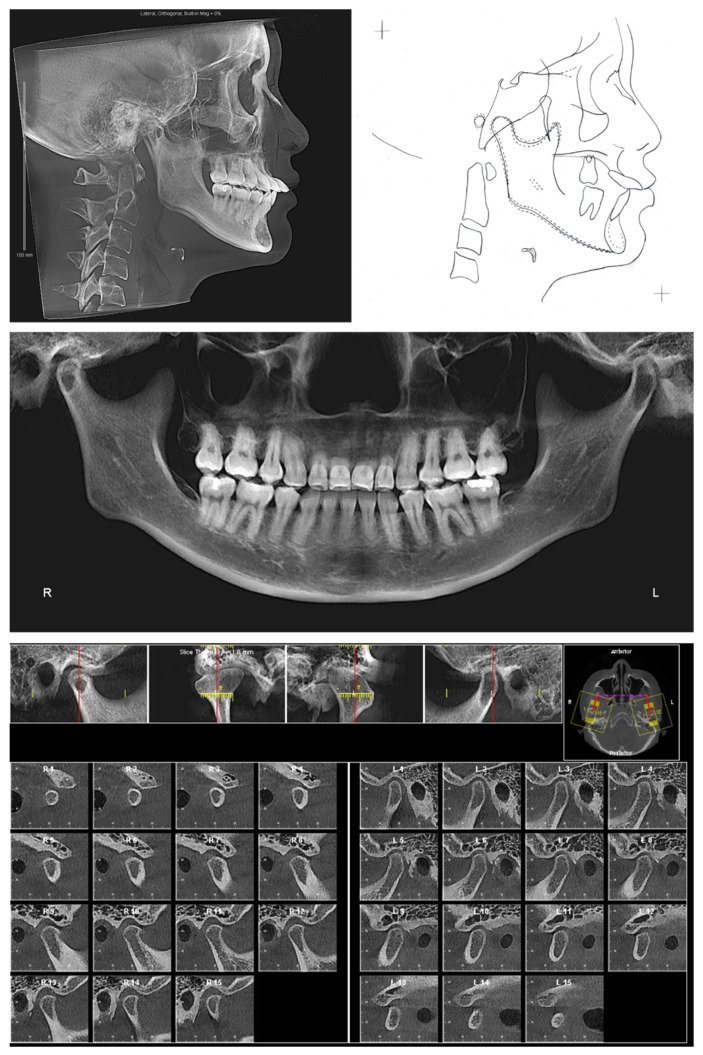
Pretreatment radiographs and cephalometric tracing.

Intraoral examination (Figure 1 and Figure 2, Table 1) showed the patient had one premolar missing in each quadrant and one lower incisor missing, which matched the patient’s previous orthodontic treatment history. Anterior-posteriorly, the patient had class III molar relationships on both sides; class I canine relationship on the right side and class III canine relationship on the left side. The maxillary incisors were severely proclined and protrusive. The mandibular incisors were normoclined and normotrusive. The overjet was 9.5 mm. Transversely, the maxillary midline was coincident to the face, and the mandibular midline was 1 mm to the left with one missing lower incisor. The curve of Wilson was mild. Vertically, the overbite was −0.5 mm with maxillary left central incisor and had bite impingement with maxillary right central incisor. The curve of Spee was 1.5 mm.

The lateral cephalogram (Figure 2, Table 1) displayed that the patient was skeletal Class III with an orthognathic maxilla. The mandible was in the orthognathic range with prognathic tendency. The patient had a mesofacial skeletal pattern.

**Figure 3:**
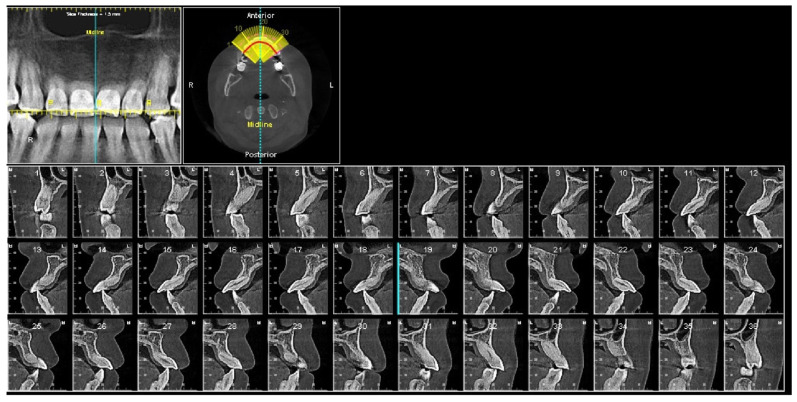
Pretreatment cross section images of maxillary anterior teeth. Maxillary right canine: slices #4–#8. Maxillary right lateral incisor: slices #9–#13. Maxillary right central incisor: slices #14–#18. Maxillary left central incisor: slice #19–#23. Maxillary left lateral incisor: #24–#28. Maxillary left canine: #29–#34.

The panoramic X-ray (Figure 2) showed that all third molars were missing. Due to the proclination of maxillary anterior teeth, the panoramic X-ray could not properly display the roots of the maxillary anterior teeth. Thus, a series of cross-sections were generated from the CBCT image. As demonstrated in Figure 3 and Table 2, the roots of maxillary incisors were penetrated out of the palatal cortical layer and presented with severe root resorption. The lengths of maxillary canine roots were acceptable, but both canines lacked palatal alveolar bone support.

The clinical exam showed the mouth opening range is within the normal limit. No pain, clicking, or crepitus was detected on either side of the temporomandibular joints. Both joints presented with a well-defined, continuous cortical layer (Figure 2). When comparing the left and right sides, the sagittal slices of temporomandibular joints (Figure 2) showed uneven joint spaces, indicating possible CO-CR discrepancy.

## 3. Treatment Objectives

The treatment objectives were to achieve (1) ideal overjet and overbite, (2) normal occlusion with Class I canine relationships on both sides, (3) stable and functional occlusion, (4) improved profile and smile esthetics, (5) minimized further root resorption, and (6) improved periodontal health by torquing the roots of maxillary anterior teeth into the alveolar ridge.

## 4. Treatment Alternatives

After a thorough explanation and discussion, the patient was well aware of the extreme proclination and root resorption with maxillary anterior teeth and missing nine permanent teeth (one mandibular incisor, four premolars, and four third molars). Due to the amount of missing teeth, no extraction could be afforded for the orthodontic treatment. Thus, a treatment plan involving TADs to en-mass retract the maxillary dentition was presented and well accepted by the patient. The patient fully understood that more root resorption probably would occur during the treatment, and she may lose the maxillary anterior teeth. Progress x-rays would be needed to closely monitor the root length.

In detail, a mandibular removable bite plate will be delivered to (1) find the CR bite, (2) eliminate the anterior occlusion trauma, (3) provide vertical clearance while retroclining the maxillary anterior teeth, and (4) find the proper amount of clockwise rotation of the mandible which would improve the skeletal sagittal relationship and profile.

In the maxillary arch, a modified TPA with bands on maxillary second molars will be used to connect to mid-palatal TAD for maxillary dentition en-mass retraction. In addition, light elastics from the maxillary incisor to mid-palatal TADs will be utilized to help correct the angulation of maxillary anterior teeth.

**Figure 4:**
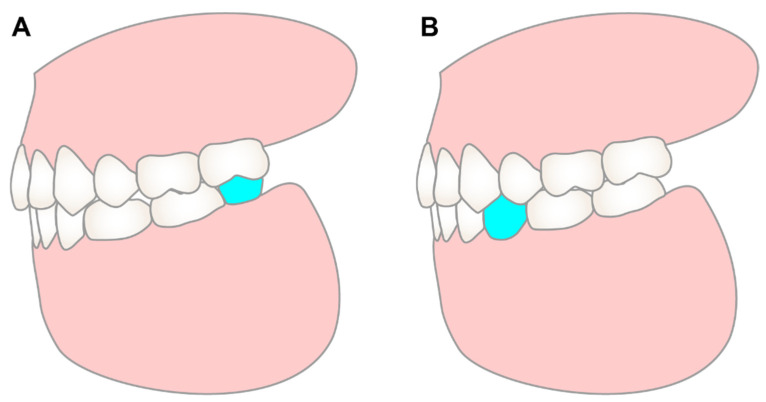
The illustration of different treatment options. Both treatment options involve the distalization of the maxillary arch and achieving class I molar and canine relationships on the right side. While for the left side, both options will use the mandibular left canine to substitute the mandibular left lateral incisor, and use the mandibular left first premolar to substitute the mandibular left canine. The difference between the two options is: option 1 (**A**) will finish in class III molar relationship on the left side, and place a retromolar implant (blue) to articulate with the maxillary left second molar; option 2 (**B**) will distalize the mandibular left molars to achieve class I molar relationship, and open space for a premolar implant (blue).

In the mandibular arch, the occlusal coverage of the mandibular second and first molar on the bite plate will be removed gradually, and short Class III vertical elastics will be delivered to extrude and distalize the mandibular molars. Once stable posterior occlusion has been established, the bite plate will be removed, and the rest of the mandibular arch will be leveled and aligned. The mandibular left canine will be used to substitute lateral incisor, and the mandibular left second premolar will be used to substitute canine. Two options were provided to set up the mandibular arch. Option one is to finish with class III molar on the left side, then the patient will need an implant restoration distal to the mandibular left second molar to articulate with maxillary left second molar. Option two is to distalize the mandibular left molars to open the space for an implant restoration of the premolar (Figure 4). The advantage of option one is short orthodontic treatment time, as long treatment time is a high-risk factor of orthodontic root resorption [9,10]. But the thin alveolar ridge distal the second molar could increase the difficulty and reduce the success rate of implant restoration. Option two is more beneficial for the implant restoration, but puts the patient at risk of more root resorption.

## 5. Treatment Progress

**Figure 5:**
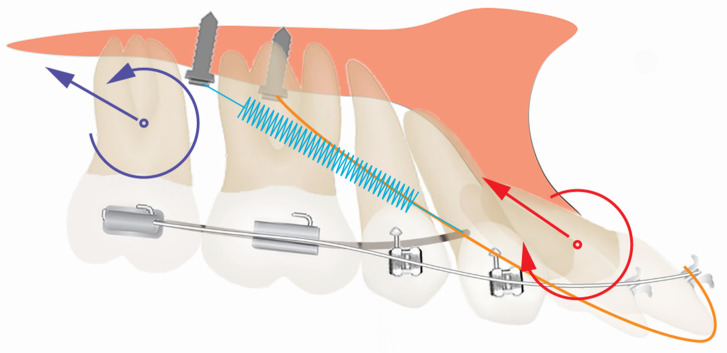
The illustration of the biomechanics for the palatal TADs for molar distalization and the correction of maxillary incisor proclination. The modified TPA (dark grey) was connected to mid-palatal TAD by NiTi coils (blue) for maxillary molar distalization. A distal, intrusion force and a counterclockwise moment were applied to the maxillary second molar (purple arrows). The purple circle represents the center of resistance of the maxillary second molar. The patient was instructed to wear 5/16 3.5 oz elastics (orange) from the lingual buttons on the maxillary central incisors to the mid-palatal TADs. A distal, intrusion force and a clockwise moment were applied to the maxillary central incisor (red arrows). The red circle represents the center of resistance of the maxillary central incisor.

A mandibular removable bite plate with occlusal coverage on mandibular molars and premolars was delivered. The height of occlusal coverage was adjusted to make sure the patient had an even bite on both sides and did not feel any discomfort in the temporomandibular joint region. The maxillary arch was bonded with 3M Unitek 0.018 MBT bracket system. The modified TPA was connected to mid-palatal TAD by NiTi coils for maxillary dentition en-mass retraction. The patient was instructed to wear 5/16 3.5 oz elastics from the lingual buttons on the maxillary central incisors to the mid-palatal TADs. The initial archwire was 0.016 × 0.022 Bioforce (Figure 5).

**Figure 6:**
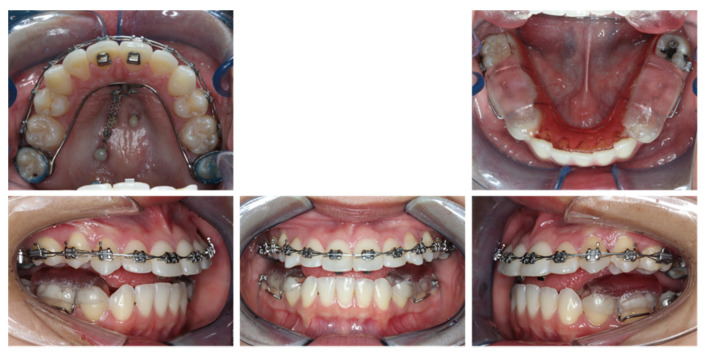
Eight-month progress facial and intraoral photographs.

Eight months into treatment (Figure 6), the retroclination of maxillary anterior teeth was observed. The posterior region of the mandibular bite plate covering the second molars was sectioned, and the mandibular second molars were bonded. The patient was instructed to wear 3/16 3.5 oz vertical elastics from mandibular second molars to maxillary second molars for mandibular second molar extrusion. Once solid occlusion contact was established with second molars on both sides, the occlusal coverage of the first molars on the mandibular bite plate was sectioned, and the mandibular first molars were banded. The patient was instructed to wear 3/16 3.5 oz Class III elastics from mandibular first molars to maxillary second molars for mandibular first molar extrusion and distalization.

**Figure 7:**
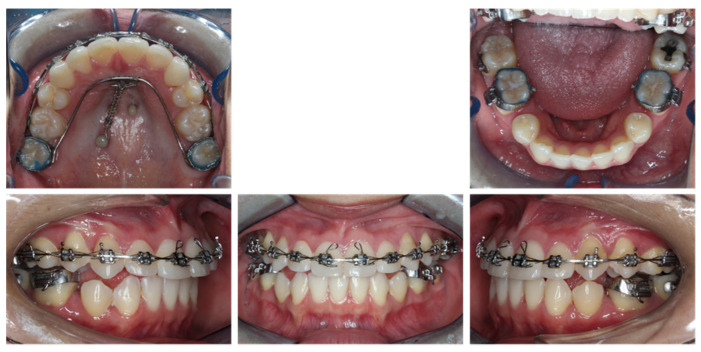
Thirteen-month progress facial and intraoral photographs.

A significant amount of mandibular molar extrusion and distalization was observed thirteen months in treatment, with space opened between the first molars and the second premolars (Figure 7). Since the occlusal contact was detected with all the molars, the mandibular bite plate was removed, and the rest of the mandibular arch was bonded to start leveling and aligning. In addition, with the retroclination of maxillary anterior teeth, occlusal interference was observed with the lingual button and mandibular anterior teeth. Thus, the lingual buttons on maxillary anterior teeth were removed, and the patient was instructed to continue anterior elastics from maxillary central incisor brackets to the mid-palatal TADs. The elastics would wrap around the incisal edge of the central incisors, which would provide a distalization and intrusion force. The maxillary archwire was changed to 0.017 × 0.025 TMA.

**Figure 8:**
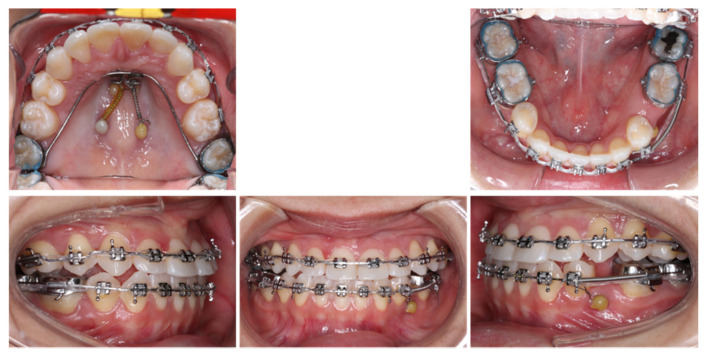
Thirty-fifth-month progress facial and intraoral photographs.

Twenty-two months into treatment, the patient informed us that due to the mouth opening limitation and the non-ideal condition of available alveolar bone distal to the mandibular left second molar, her dentist would like to have us open an implant space in the premolar region rather than an implant distal to the mandibular second molar. A thorough discussion about the risk of prolonged orthodontic treatment was conducted again with the patient. The necessity of additional TAD in the mandibular arch was also explained to the patient. The patient selected and well-accepted the option to open the premolar space. Thus, a TAD was placed distal to the mandibular left second premolar for mandibular left molar distalization (Figure 8). The patient was also instructed to wear Class III elastics to achieve Class I canine relationship on both sides. And the patient was debonded Forty-three months after starting the orthodontic treatment. Due to COVID-19, the treatment was paused for six months, so the total active treatment time was thirty-seven months.

After debonding, a palatal fixed retainer overlying with a removable wrap-around retainer were delivered as the retainers of the maxillary arch. The wrap-around retainer was fabricated with labial acrylic plate over maxillary canine to canine. For the mandibular arch, a removable Hawley retainer was delivered. The patient was instructed to wear the removable retainers full time for the first 6 months after debonding, and then nighttime only.

## 6. Results

**Figure 9:**
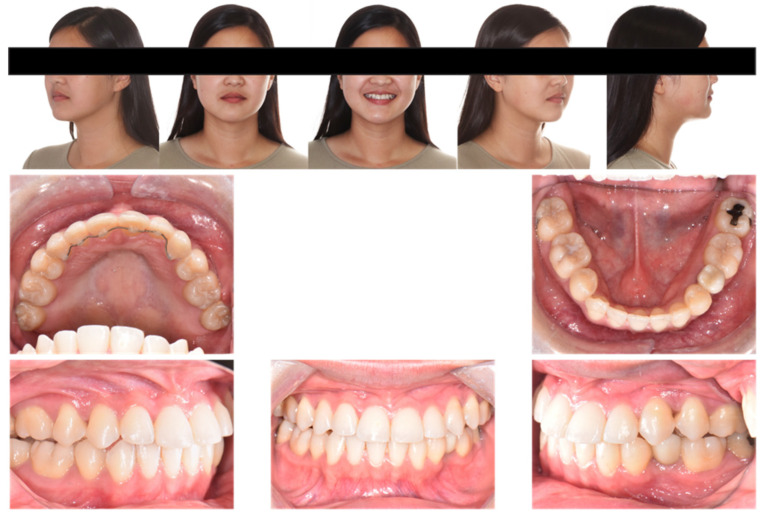
Posttreatment facial and intraoral photographs.

**Figure 10:**
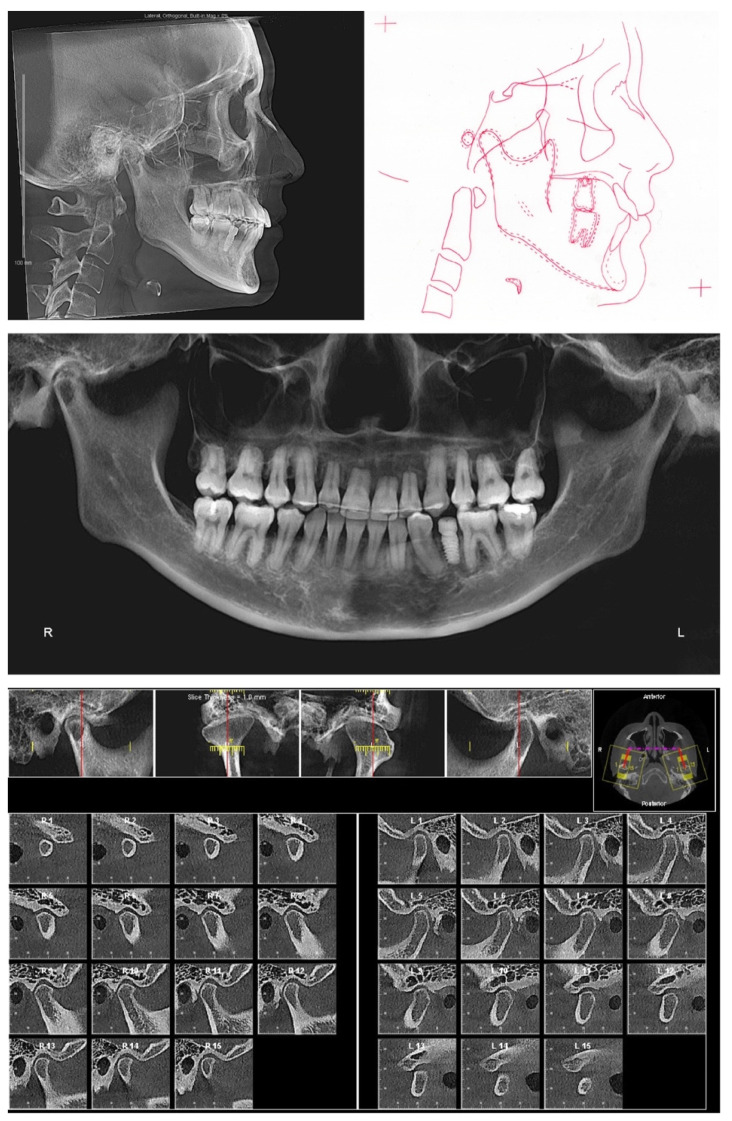
Posttreatment radiographs and cephalometric tracing.

**Figure 11:**
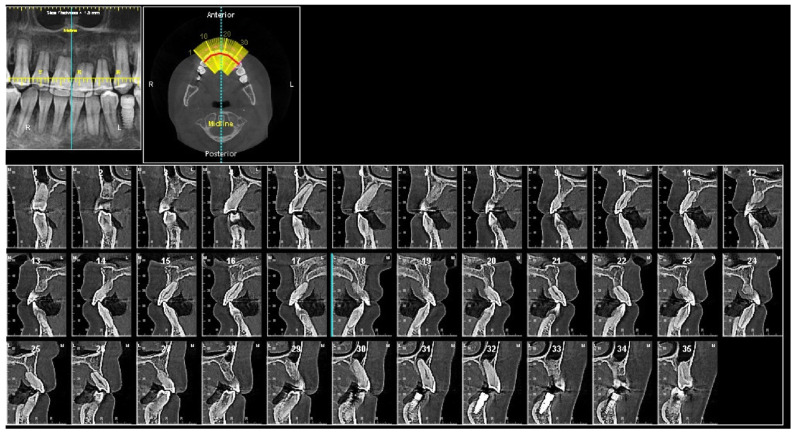
Posttreatment cross section images of maxillary anterior teeth. Maxillary right canine: slices #3–#7. Maxillary right lateral incisor: slices #8–#12. Maxillary right central incisor: slices #13–#17. Maxillary left central incisor: slice #18–#23. Maxillary left lateral incisor: #24–#28. Maxillary left canine: #29–#33.

At the completion of treatment, the severe proclination and protrusion of maxillary anterior teeth were corrected, and acceptable overbite and overjet were established (Figure 9 and Figure 10). The temporomandibular joint spaces were similar on both sides, indicating a CR occlusion was established (Figure 10), and the patient didn’t report any joint discomfort during and at the end of the treatment. The intact cortical layers of the condylar heads were also maintained (Figure 10). The cross-section slices of the maxillary anterior teeth further proved the correction of the inclination of the anterior teeth and a better position of the tooth roots in relation to the alveolar ridge (Figure 11). When comparing the root length of maxillary anterior teeth, minimum root resorption (within 1 mm) was observed during the whole treatment (Table 2).

**Figure 12:**
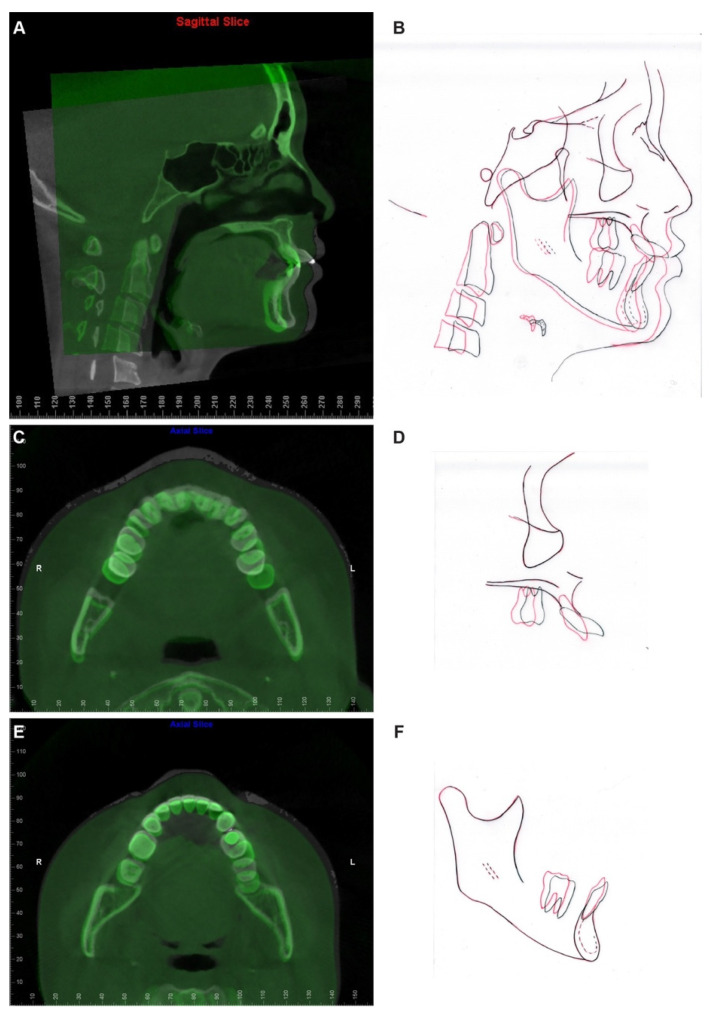
CBCT and cephalometric superimposition. (**A**) The sagittal slice at the maxillary left central incisor level of pre- (white) and post- (green) treatment CBCT images superimposed based on cranial base. (**B**) The superimposition of pre- (black) and post- (red) treatment cephalometric tracings superimposed based on cranial base. (**C**) The Axial slice of pre- (white) and post- (green) treatment CBCT images superimposed based on the cranial base. The slice was captured at the level of the cervical region of maxillary anterior teeth to demonstrate the amount of maxillary molar distalization achieved during treatment. (**D**) The superimposition of pre- (black) and post- (red) treatment cephalometric tracings of the maxilla. (**E**) The Axial slice of pre- (white) and post- (green) treatment CBCT images superimposed based on mandibular symphysis. The slice was captured at the level of the cervical region of mandibular anterior teeth to demonstrate the amount of mandibular molar distalization achieved during treatment. (**F**) The superimposition of pre- (black) and post- (red) treatment cephalometric tracings of the mandible.

Pre- and post-treatment superimposition (Figure 12, Table 1) demonstrated the maxillary and mandibular molar distalization and anterior teeth retraction and retroclination. In detail, the maxillary central incisors were retracted 7.9 mm and retroclined 33 degrees (Figure 12A,B), the maxillary left molars were distalized 8 mm, the maxillary right molars were distalized 4 mm (Figure 12C), the mandibular left molars were distalized 5 mm, and the mandibular right molars were distalized 2 mm (Figure 12E). It is worthy to note that the molar distalization in this case has reached its anatomic limitation, as in the post-treatment panoramic X-ray (Figure 10), a limited amount of maxillary tuberosity was left distal to the maxillary left second molar, and the mandibular left second molar was position close to the retromolar fossa.

Overall, the smile esthetics was improved, and the patient was satisfied with the outcome.

## 7. Discussion

Different types of TADs supported molar distalizers have been reported [6,11,12,13] with the average amount of molar distalization as 4.07 mm when midpalatal TADs were used [13]. In the current case, by utilizing an easy mechanical setup composed of a modified TPA, mid-palatal TAD, and NiTi Coils, 8 mm distalization was achieved on the right side and 4 mm distalization was achieved on the left side with proper molar root angulation. With only one or two TADs needed in the mechanic setup and no special patented appliance required, the current case presented an easy, efficient, and less invasive way for maxillary molar distalization. However, further studies are warranted to compare the distalization efficacy and efficiency of the current mechanical setup with other TADs supported molar distalizers.

In the current case, we identified that molar distalization had reached its anatomic limitation based on the evaluation on X-rays. Identifying the limit of orthodontic tooth movement has been a long-lasting topic. Moving teeth out of alveolar bone support would lead to irreversible periodontal and dental damage [14,15]. It was believed that, during orthodontic tooth movement, maxillary alveolar bone has better remodeling property than mandibular bone as it has lower bone density than the mandible. Thus, multiple orthodontic treatment philosophies use the mandibular arch as the orthodontic treatment objective reference to set up the mandibular anterior teeth to the center of the alveolar bone housing, and match the maxillary arch to the mandibular arch to get ideal overbite and overjet [16]. However, cases with difficulty in upper anterior teeth retraction, torque correction, and root resorption have been recently reported [17,18]. It is worth noting that the root contact with the labial or palatal cortical plate at the root apex level during orthodontic tooth movement has been associated with root resorption [19]. In addition, Vardimon et al. reported that, when retracting maxillary incisors, the ratio between palatal cortical bone remodeling and tooth movement is only 1:2, meaning that the alveolar bone remodeling is significantly delayed compared to the rate of orthodontic tooth movement [20]. It is not difficult to imagine that, even a great amount of palatal bone presented before the orthodontic treatment, if the treatment objective is to retract the anterior teeth out of the boundary of the alveolar bone, the cortical bone remodeling rate could not catch up with the tooth movement rate; instead, it will end up with collision between the tooth root and cortical bone plate, then leading to root resorption and periodontal defect. In the current case, the patient presented with severe proclined maxillary anterior teeth after two rounds of orthodontic treatment. The current pre-treatment CBCT revealed severe root resorption and penetration of root apex out of the palatal cortical bone layer, which corroborated the correlation between cortical plate proximity and apical root resorption. In addition, the shortest root was detected with the maxillary left central incisor. A cross-section image along the long axis of the maxillary left central incisor revealed the contact between the root apex and the buccal wall of the incisive canal. Recent studies showed that root contacting the incisive canal could cause external apical root resorption [21,22]. Since the dimension and position of the incisive canal could not be evaluated on two-dimensional X-rays, three-dimensional CBCT images would be necessary to evaluate the limitation of orthodontic tooth movement when a large amount of tooth retraction is planned.

Previous studies reported that proper orthodontic therapies torquing the buccally positioned tooth roots back to the alveolus could improve periodontal health by reducing intraosseous defects or furcation lesions [23,24] and, consequently, bone regeneration [25]. In the current case, palatal bony support could be detected with maxillary canines and right lateral incisor, but not the maxillary central incisors and left lateral incisor on the post-treatment CBCT even after 33 degrees of angulation correction. Unfortunately, a thinner alveolar ridge was presented post-treatment compared to that in the pre-treatment image. The palatal cortical bone plate responded to orthodontic correction differently compared to the buccal cortical bone. Further investigation is required to explain this observation. Possible explanations may be related to the existing keratinized, highly dense, and tensile specialized palatal mucosa. Additionally, palatal anatomy, such as the morphology of the palatal vault as well as the size and proximity of the incisive canal, may also need to be considered.

Last but not the least, orthodontic root resorption is one of the most frequently reported side effects of orthodontic movement [9,10]. Orthodontic root resorption is related to various factors, while long orthodontic treatment time and a large amount of apical displacement pose great risk factors for root resorption [9,10]. Sameshima et al. [10] stated that a tooth with a short root has a favorable long-term prognosis and needs not to be extracted and replaced by restoration. Thus, every effort was provided to reserve the maxillary anterior teeth in the current case. However, there is still no clear clinical guideline on how to orthodontically manage the cases with a previous history of root resorption, except by using light forces and period radiographic evaluation [7,10]. In the current case, the maxillary archwire was maintained in 0.016 × 0.022 Bioforce until proper tooth angulation was achieved. In addition, the mandibular bite plate was fabricated without anterior coverage to avoid occlusal contact on the maxillary anterior teeth during treatment. The root lengths of maxillary anterior teeth were monitored periodically with X-rays during the whole treatment. At the end of treatment, minimum root resorption was detected after a great amount of anterior teeth retraction, torque correction, and intrusion. In summary, the maxillary anterior teeth with severe root resorption were preserved esthetically and functionally.

## 8. Conclusions

In conclusion, we described a case with successful en-mass distalization to correct the severe proclined maxillary anterior teeth. Overall, the patient’s profile has been improved, and functional and stable occlusion has been achieved. The findings from this case indicate that with proper mechanics setup, a great amount of tooth movement can be accomplished without compromising the previously resorbed roots. On the other hand, the initial records of the patient showed that improper diagnosis and treatment could lead to irreversible damage to the patient’s oral health. Therefore, the periodontal limitation of tooth movement should be carefully evaluated during the whole orthodontic treatment.

## Figures and Tables

**Table 1:** Cephalometric analysis. Asian norms were used as the reference.

Measurement	Average ± SD	Before Treatment	After Treatment	Change
SNA (∘)	82.8 ± 4.0	82.4	82.7	0.3
SNB (∘)	80.1 ± 3.9	83.2	81.1	−2.1
ANB (∘)	2.7 ± 2.0	−0.8	1.6	2.4
FH to NPo (∘)	87.4 ± 3.0	91.4	89.3	−2.1
MP to FH (∘)	29.1 ± 4.8	26.5	28.7	2.2
MP to SN (∘)	32.5 ± 5.2	32.6	34.8	2.2
*Y*-axis (∘)	65.8 ± 3.1	56.2	58.0	1.8
U1 to L1 (∘)	125.4 ± 7.9	85.1	121.2	36.1
U1 to SN (∘)	105.7 ± 6.3	148.1	115.5	−32.6
U1 to NA (∘)	23.6 ± 4.6	65.7	32.7	−33
U1 to NA (mm)	4	14.2	6.3	−7.9
L1 to MP (∘)	94.7 ± 5.2	94.2	88.5	−5.7
L1 to NB (∘)	30.8 ± 4.9	30.1	24.5	−5.6
L1 to NB (mm)	7	5.6	4.7	−0.9
Upper lip to E-plane (mm)	−3.7 ± 2.0	−1.8	−4.2	−2.4
Lower lip to E-plane (mm)	−2.0 ± 2.0	−0.8	−3.1	−2.3

“∘” stands for “degrees”, “mm” stands for “millimeter”.

**Table 2:** Maxillary anterior teeth root length measured on CBCT.

	Before Treatment (mm)	After Treatment (mm)	Change (mm)
Maxillary left canine	15.4	14.4	−1.0
Maxillary left lateral incisor	9.2	8.3	−0.9
Maxillary left central incisor	5.6	5.2	−0.4
Maxillary right central incisor	8.8	8.4	−0.4
Maxillary right lateral incisor	8.5	8.2	−0.3
Maxillary right canine	15.9	15.4	−0.5

## Data Availability

Not applicable.
